# Unbiased Determination of Adsorption Isotherms by Inverse Method in Liquid Chromatography

**DOI:** 10.3390/molecules28031031

**Published:** 2023-01-19

**Authors:** Szabolcs Horváth, Diána Lukács, Evelin Farsang, Krisztián Horváth

**Affiliations:** 1 CHEMARK Ltd., Peremarton Gyártelep P.O.B. 31., H-8182 Berhida, Hungary; 2 Research Group of Analytical Chemistry, University of Pannonia, Egyetem Utca 10, H-8200 Veszprém, Hungary

**Keywords:** preparative liquid chromatography, isotherm determination, spline fitting, Martin-Synge algorithm

## Abstract

The Inverse Method is a widely used technique for the determination of adsorption isotherms in liquid chromatography. In this method, isotherm is determined from the overloaded peak profile of the component by the iterative solution of the mass balance equation of liquid chromatography. Successful use of this method requires a prior assumption of equation of isotherm (Langmuir, BET etc.). In this work, we have developed an inverse method that gives results of similar accuracy to the frontal analysis without assuming the equation of the isotherm. The oversaturated peaks were calculated using a spline fitted to data points instead of the derivative of the isotherm. The distribution of the isotherm points were optimized for minimizing the difference between the measured and calculated overloaded peaks. The accuracy of the developed method was verified with synthetic benchmark peaks and by the determination of isotherm of buthyl-benzoate under real conditions. The results confirmed that the accuracy of the developed method is similar to that of Frontal Analysis.

## 1. Introduction

For carrying out fast and effective separations, it is important to know the thermodynamic processes that affect the separation [1]. In preparative chromatography, the optimal separation conditions and the loadability of the column depends primarily on the type of the adsorption isotherm [2,3]. Estimation of adsorption isotherms also give deeper understanding of the separation processes and its molecular interactions [4].

The classification of adsorption isotherms is often based on their shapes [5,6]. Type-I isotherms (Langmuir or similar) are convex functions with a horizontal asymptote equal to the surface capacity. Type-III adsorption isotherms (BET or similar), sometimes called anti-Langmuir, on the other hand are concave with a vertical asymptote. In terms of energy distribution, the Langmuir model is unimodal and the bi-Langmuir is heterogeneous bimodal [7].

Numerous methods are available for the determination of adsorption isotherms. Five direct chromatographic methods are available for this purpose: frontal analysis (FA) [8], frontal analysis by characteristic point (FACP) [9], elution by characteristic point (ECP) [10], pulse methods (e.g., elution on a plateau or step and pulse method) [11], and the retention time method (RTM) [12]. In case of Frontal Analysis (FA), the solutions of the compound with increasing concentration are injected to the column. For each concentration, a breakthrough curve is determined. The weight of the adsorbed solution is determined from the retention volume of the inflection point of the breakthrough curve. The method that is traditionally considered to be the most accurate for the determination of adsorption isotherms is the FA, and it can be used regardless of the type of the adsorption [13].

A fast and efficient method for determination of adsorption isotherm is the so called Inverse Method (IM) [14]. In case of the IM, the isotherm is derived from the overloaded elution profile of the compound by the iterative solving of the mass balance equation of the liquid chromatography. For the successful application of this method, a presumption is needed in matter of the type of the isotherm. If we use Langmuir equation in the method, the inverse method will inevitably end up with a Langmuir equation, even if the stationary phase itself contains two or three adsorption groups of different energies. That is, the component is in fact a bi- or tri-Langmuir isotherm, respectively. This is particularly problematic when the component has a rare type of isotherm. Accordingly, inverse method can be called a biased method [15,16,17].

The idea of using interpolation instead of a closed adsorption isotherm model has previously been investigated by some authors. Haghpanah et al. used the Transport Dispersive model with a Linear Driving Force mass transfer model and estimated adsorption isotherms by the Inverse Method (IM) with a Sequential Quadratic Programming algorithm [18]. Stineman interpolation was used, which found to be significantly advantageous over linear interpolation [19]. The advantage of Stineman interpolation is the fewer required segments to estimate a nonlinear function in contrast to linear interpolation. Fornstedt et al. also developed a modified IM that, instead of fixed adsorption isotherm models, uses monotone piecewise interpolation. They have shown that it might not be possible to find a closed adsorption isotherm model that account the inflection points in case of complicated isotherms [20].

Numerical isotherm estimation methods have also attracted considerable attention in preparative chromatography. Gao et al. studied the possibility of using neural networks to describe the isotherms. Isotherm derivatives are generated as outputs of neural networks. The neural network can represent any form of isotherm by increasing the number of neurons in the hidden layer. Simulations and experiments demonstrated that the proposed neural network isotherm model can give a good estimation of adsorption isotherms from chromatograms [21].

Although frontal analysis is a highly accurate method for isotherm determination, it requires a large amount of material and solvent. In contrast, the inverse method requires little material, but the isotherm equation must be known beforehand, accordingly these methods are biased inherently. The aim of this work was to present a model-free-unbiased inverse method for the determination of adsorption isotherms with the same accuracy as frontal analysis, and with the low material requirements of inverse methods.

## 2. Theory

### 2.1. Calculation of Elution Profiles

In this work, elution profiles were calculated by solving the equilibrium-dispersive (ED) model with the Martin-Synge algorithm [22]. It was shown that, when the mass transfer kinetics is fast and when the dispersion coefficient of the solute can be calculated accurately, the differential mass balance of the solute [23,24,25] can be written as:

(1) $$\frac{\partial \;c[z,t]}{\partial \;t}=-F\frac{\partial \;q[z,t]}{\partial \;t}-u_{0}\frac{\partial \;c[z,t]}{\partial \;z}+D_{\mathrm{app}}\frac{\partial^{2}\;c\left[z,t\right]}{\partial \;z^{2}}$$

where *q* and *c* are the concentration of the solute on the stationary and in the mobile phases, respectively, *t* and *z* are time and spatial variables, respectively, $u_{0}$ the linear velocity of the eluent, and F=(1/ϵ)/ϵ is the phase ratio of the column, where ϵ is the total column porosity of the column. The isotherm, *q*, is a function of *c*, q=f(c).

The apparent dispersion coefficient, $D_{\mathrm{app}}$ can be approximated as:

(2) $$D_{\mathrm{app}}=u\;\frac{H}{2}$$

where *H* is the height equivalent to a theoretical plate. This approximation allows the equilibrium-dispersive model to correctly take into account the influence of the column efficiency on the profile of elution bands.

Assuming that the column is divided into *M* vessels, Equation (1) can be transformed into a series of ordinary differential equations (ODE) that are continuous in time but discrete in the spatial variable [22]. For the *m*th vessel, the following ODE is defined:

(3) $$\frac{\partial \;c_{m}\left[t\right]}{\partial \;t}=-F\frac{\partial \;q_{m}\left[t\right]}{\partial \;t}-u_{0}\frac{\partial \;c_{m}\left[t\right]-c_{m-1}\left[t\right]}{\Delta \;z}$$

where Δz is the length of a vessel (Δz=L/M), and 1≤m≤M. $c_{m}$ and $c_{m-1}$ are the concentrations of the solute in the *m*th and (m−1)th vessels.

The term $\frac{\partial \;q_{m}\left[t\right]}{\partial \;t}$ in Equation (3) can also be expressed as:

(4) $$\frac{\partial \;q_{m}\left[t\right]}{\partial \;t}=\frac{d\;q_{m}}{d\;c}\frac{\partial \;c_{m}\left[t\right]}{\partial \;t}$$

Substituting this into Equation (3) gives the final formula:

(5) $$\frac{\partial \;c_{m}\left[t\right]}{\partial \;t}=-\frac{u}{1+F\frac{d\;q_{m}}{d\;c}}\frac{\partial \;c_{m}\left[t\right]-c_{m-1}\left[t\right]}{\Delta \;z}$$

The initial condition for m>1 is $c_{m}\left[t=0\right]=0$, while the injection profile in case of rectangular injection is $c_{1}\left[0<t\leq t_{inj}\right]=c_{inj}$ with $c_{inj}$ the injected concentration, and $t_{inj}$ the injection time. The elution profile of the solute is the solution of the last vessel, $c_{M}$.

In Equation (2) it was shown that, according to the ED model, the apparent dispersion coefficient, $D_{\mathrm{app}}$, is equal to u(H/2). Thus, Equation (5) is equivalent to the equation of the ED model if Δz=H, or in other words, if the number of slices into which the column is divided is equal to the number of its theoretical plates:

(6) $$\Delta z=\frac{L}{N}$$

where *L* is the length of the column, and *N* the number of theoretical plates.

### 2.2. Models of Adsorption Isotherms

Solution of the Equilibrium-Dispersive model, Equation (1), requires an isotherm model. The most commonly used model is isotherms in chromatographic modeling are the Langmuir, bi-Langmuir and BET isotherms.

The Langmuir isotherm is a two-parameter isotherm model. Accordingly, it is always possible to determine a Langmuir isotherm that is tangent to any complex isotherm model at the origin and has the same curvature around the origin (provided the model is convex upward). The Langmuir adsorption isotherm is conventionally written as:

(7) $$q=\frac{b_{s}\;q_{s}\;c}{1+b_{s}\;c}$$

where $q_{s}$ is the saturation capacity of the stationary phase, *q* and *c* are the concentrations of the solute on the stationary and in the mobile phases and $b_{s}$ is the adsorption equilibrium constant of the compound.

In many cases the surface of the adsorbent used for chromatographic separations is not homogeneous. The simplest model for a nonhomogeneous surface is a mixed (patchwork) surface covered with patches made of two different homogeneous surfaces, i.e., covered with two different kinds of chemical groups. The bi-Langmuir isotherm equation:

(8) $$q=\frac{b_{s,1}\;q_{s,1}\;c}{1+b_{s,1}\;c}+\frac{b_{s,2}\;q_{s,2}\;c}{1+b_{s,2}\;c}$$

where $b_{s,1}$ and $b_{s,2}$ are the adsorption equilibrium constants of the two different adsorption centers and $q_{s,1}$ and $q_{s,2}$ are the saturation capacity of the two different adsorption centers.

The BET isotherm model assumes that the solute molecules can adsorb from the solution onto either the bare surface of the adsorbent or a layer of solute already adsorbed. The BET isotherm may be represented by the following equation:

(9) $$q=\frac{q_{s}\;b_{s}\;c}{\left(1-b_{l}\;c\right)\;\left(1-b_{l}\;c+b_{s}\;c\right)}$$

where $q_{s}$ is the saturation capacity of the stationary phase, *q* and *c* are the concentrations of the solute on the stationary and in the mobile phases, $b_{s}$ and $b_{l}$ are equilibrium constants of adsorption on the surface of the stationary phase and on the adsorbed layer of solutes [1].

### 2.3. Determination of Isotherms by the Inverse Method

Isotherm of solutes are usually determined by batch experiments that requires a large amount of sample compound and produces a lot of waste. By using inverse method, one can determine the isotherm from overloaded peak profiles by fitting the solution of the ED model on the measured peak. However, in that case, the analyst should guess an isotherm model of the solute. In our developed method, a B-spline fitted to a number of data points is used as the derivative of isotherm in the ED model (see Equation (5)) during the application of inverse method. The values of isotherm points are optimized in order to minimize the difference between the measured and calculated peak profiles. This difference is expressed by the sum of square residuals, which can be calculated as follows:

(10) $$\mathrm{SSR}=\sum_{i}{\left(c_{i}^{\mathrm{calc}}-c_{i}^{\mathrm{meas}}\right)}^{2}$$

where $c_{i}^{\mathrm{calc}}$ and $c_{i}^{\mathrm{meas}}$ are the measured and calculated concentrations at point *i* respectively.

## 3. Results and Discussion

### 3.1. Distribution of Isotherm Points

For the success of the developed isotherm-equation-free method, it is crucial to determine the minimum number of isotherm points and their distribution along the *x* axis that provides accurate band profiles. By varying the number and distribution of isotherm points band shapes can be calculated by the isotherm-equation-free Martin-Synge algorithm. These can be compared to the band shape calculated from the isotherm equation used for the generation of isotherm points. The accuracy of the different cases can be quantified by the sum of square residuals of these band profiles. During our calculations, the number of isotherm points were varied between 10 and 500. For the distribution of the isotherm points, five different scenarios were studied. These were the following:

For Langmuir isotherm:

1 linear distribution in the abscissa,
2 logarithmic distribution in the abscissa.

For BET isotherm:

3 linear distribution in the abscissa,
4 logarithmic distribution in the abscissa,
5 logarithmic distribution in the ordinate.

**Figure 1:**
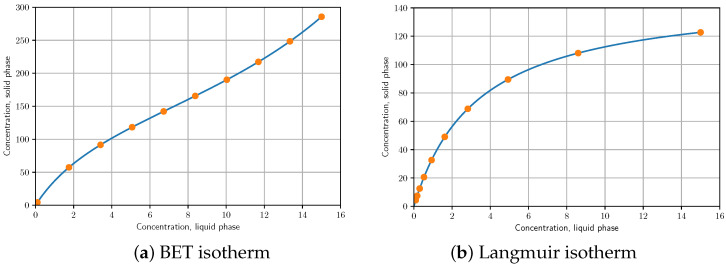
Optimal distribution of isotherm points for convex (**a**) and concave isotherm (**b**).

The SSR values calculated for the different scenarios are shown in Table 1. Close examination of the SSR values shows that for concave isotherms such as Langmuir isotherms, the logarithmic distribution of isotherm points on the ordinate gives better results, while for convex isotherms such as BET isotherms, the linear distribution of isotherm points on the ordinate gives the most accurate results (see Figure 1). It can also be concluded that the unbiased model is suitable for the calculation of overloaded peak profiles for both convex and concave isotherms, even from a small number (10–20) of isotherm points.

### 3.2. Method Verification by Benchmark Isotherm

In this section, the isotherm of a synthetically generated peak was determined by the unbiased Inverse Method. The type of the isotherm belonging to the synthetic peak was a bi-Langmuir isotherm. Calculation parameters were the following:

- saturation capacity of the first and second type of adsorption centres, $q_{s1}=150,$$q_{s2}=10$
- adsorption equilibrium constants of the two different adsorption centers, $b_{s1}=0.3,$$b_{s2}=3$
- injection time, $t_{inj}=5$ min
- injected concentration, $c_{inj}=15$
- columns length, L=10 cm
- linear velocity, $u_{0}=10$ cm/min
- phase ratio, F=0.65

The isotherm belonging to the synthetic peak was determined by the unbiased, isotherm equation-free method. Since the method requires an initial guess of the isotherm, in the first step an initial isotherm was determined using the traditional inverse method based on the isotherm equation. In the method, a Langmuir isotherm was assumed, based on the knowledge of the generated peak shape, whose parameters were determined using an inverse method. The resulting parameters of the initial guess isotherm were $q_{s}=158.61$, and $b_{s}=0.335$. 20 initial isotherm points were generated from the derivative of the Langmuir isotherm, and the values of the derivative points were changed by the simplex algorithm in order to minimize the difference between the calculated and the original (simulated) peaks. The spline was fitted to the derivative points, and this spline was used directly in the Martin-Synge algorithm (Equation (5)). The optimization process was continued until the fit between the two peaks was satisfactory, in other words, the SSR reached its minimum value. From the derivative points, the isotherm was generated by spline integration.

**Figure 2:**
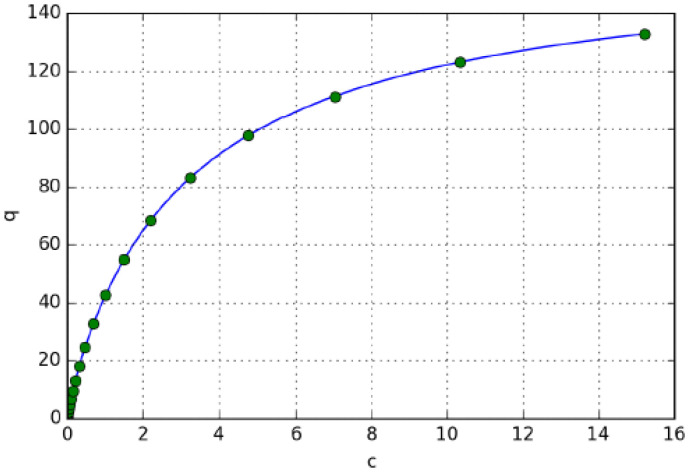
Comparison of the original bi-Langmuir isotherm (blue line) and the isotherm points determined by the inverse method (green dots).

Figure 2 shows the isotherm points determined by the developed method together with the original bi-Langmuir isotherm. It can be seen that the isotherm points determined by the unbiased inverse method fit perfectly to the bi-Langmuir isotherm. The method is suitable for isotherm determination through the optimization of the derivative of the isotherm points.

**Figure 3:**
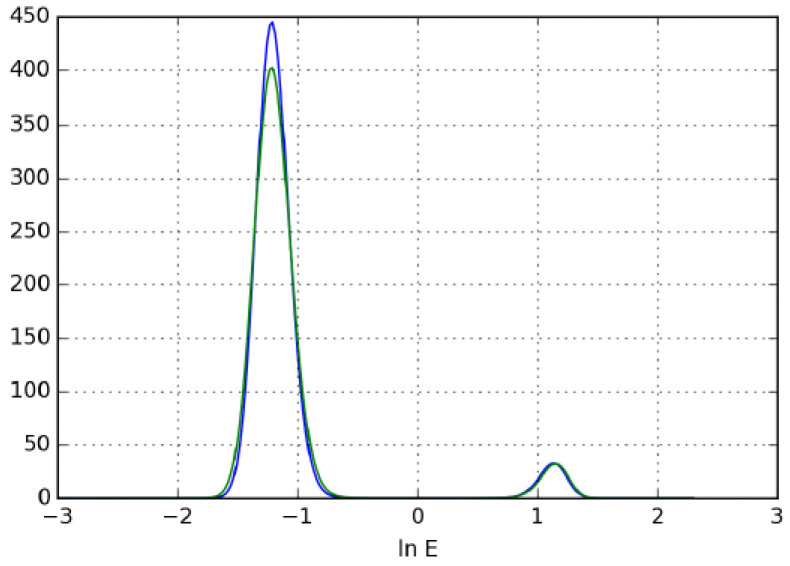
The distributions of adsorption energy belonging to the original (blue) and the calculated (green) isotherm.

The validity of the developed method was also verified by determining the adsorption energy distributions (AED) [26]. The determination of adsorption energy distribution is based on an expectation maximization algorithm. A detailed description of the method can be found in Ref. [26]. Figure 3 shows that the AED determined from the original benchmark and the calculated isotherms match almost perfectly. Both AED diagrams show two adsorption sites, confirming that the isotherm points determined by the developed inverse method indeed represent a bi-Langmuir isotherm that is identical to the original reference isotherm. It is important to note that the initial guess of the isotherm was a one-site Langmuir isotherm. Nevertheless, the developed method resulted in a bi-Langmuir isotherm, i.e., the initial estimate is independent of the shape of isotherm to be determined. Therefore, the method can be described as unbiased. The initial isotherm is only needed to reduce the time required for the calculations.

### 3.3. Determination of Butyl-Benzoate Isotherm

#### 3.3.1. Isotherm Determination by Frontal Analysis

**Figure 4:**
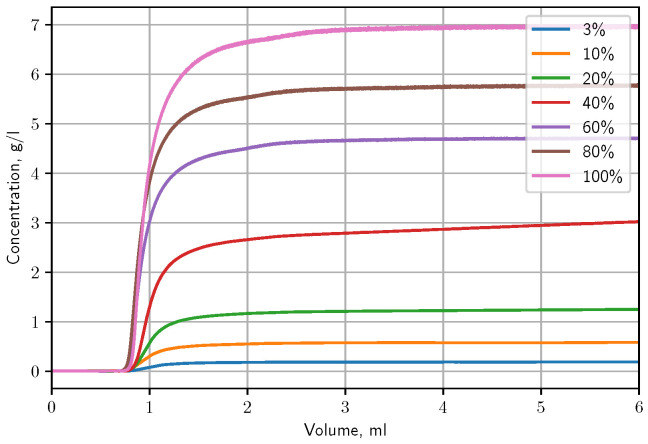
Breakthrough curves of butyl-benzoate samples at different concentration levels.

The first step of frontal analysis is the determination of breakthrough curves. Different mixtures with increasing concentrations were injected into the column by pump and for each concentration a breakthrough curve was obtained. The measurements was carried out 0.2 mL/min flow rate and 30 °C. The eluent was 65:35 methanol–water mixture. The concentration of butyl-benzoate dissolved in the mobile phase was 7.2528 g/L. During the recording of the breakthrough curves, the eluent-sample ratio was changed step-by-step, producing breakthrough curves at different butyl-benzoate levels. In Figure 4, these breakthrough curves can be seen. Note, that these breakthrough curves served as the basis of detector calibration in order to convert the detector signal (absorbance) to concentration.

The isotherm points were calculated as

(11) $$q=\frac{c\;(V_{infl}-V_{0})}{V_{a}}$$

where *c* is the concentration of butyl-benzoate, $V_{infl}$ the retention volume of the inflexion point of the front of breakthrough curve, $V_{0}$ the dead volume, and $V_{a}$ the volume of the stationary phase.

The inflection points were determined by fitting a spline to each of the breakthrough curves, and the maximum of the derivatives of the splines gave the inflection points. Dead volume was determined by injecting uracil.

#### 3.3.2. Determination of Butyl-Benzoate Isotherm by Unbiased Inverse Method

**Figure 5:**
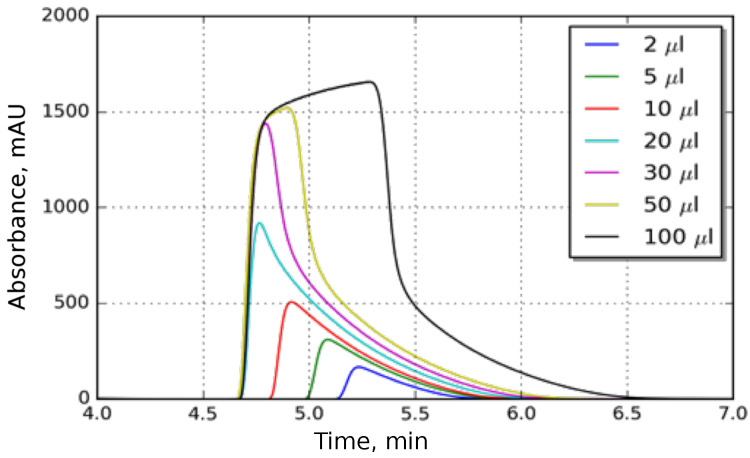
Peak shapes for the different injection volumes at 30 °C.

Figure 5 shows the chromatograms of butyl benzoate injected at different volumes. The peak shapes change significantly with the increasing injection volume. Up to 30 μL injection volume, it could be assumed that the adsorption of butyl benzoate is governed by a Langmuir-type isotherm. However, as the injection volume is further increased, it becomes apparent that the component has a BET isotherm, i.e., the adsorption is multilayered. Accordingly, during the unbised inverse method, the distribution of isotherm points were linear in the abscissa. For the determination of the isotherm, the chromatogram of the highest volume injection was used. The absorbance were converted to concentration by the calibration curve obtained at the frontal analysis.

**Figure 6:**
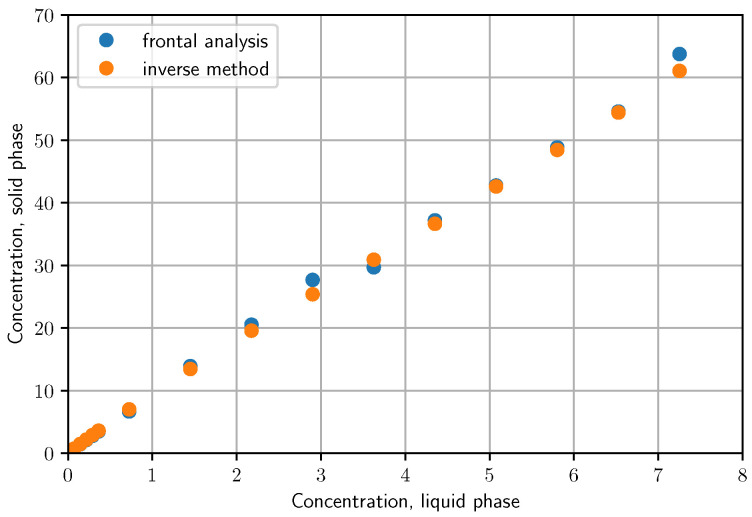
Isotherm points determined by the Frontal Analysis (blue) and by the unbiased Inverse Method (orange).

In Figure 6, the comparison of the isotherm points obtained by the Frontal Analysis and the unbiased Inverse Method can be seen. Compared to the unbiased inverse method, 3–4 points of the isotherm obtained by the frontal analysis are out of line. Apart from this, it can be seen there is sufficient agreement between the results of the two methods. The difference between the isotherms determined by the two methods is not significant. It can be concluded that the unbiased inverse method is suitable for the accurate determination of isotherms from overloaded chromatographic peaks using much less material and solvent than the frontal analysis.

## 4. Materials and Methods

### 4.1. Instrumentation and Materials

The chromatographic experiments were carried out using an HP 1100 Series liquid chromatograph (Hewlett Packard, now Agilent Technologies, Palo Alto, CA, USA), equipped with a multisolvent delivery system, an automatic injector, a column thermostat a DAD detector, and an HP Chemstation data aquisition system. Band profiles of butyl benzoate were recorded at 290 nm.

The column used during the experiments was a 50 mm × 2.1 mm Waters Cortecs C18 column packed with 5 μm particles. 65:35 methanol-water mixture was used as the eluent.

### 4.2. Computation

The numerical calculations were carried out with a software written in house in Python programming language (v. 3.8, Anaconda Python Distribution), using the NumPy and SciPy packages. In the developed, isotherm-equation-free inverse method, the derivative of isotherm is replaced by a B-spline fitted to individual data points. The sum of square difference of measured and calculated band profiles is minimized by optimizing the position of these data points individually by downhill (Neldear-Mead) simplex method [27]. The initial values of the isotherm points are determined by a classical inverse method assuming Langmuir isotherm. The number and distribution of isotherm points depend on the shape of measured band profile (see Section 3.1).

## 5. Conclusions

Knowledge of the isotherm of the components is necessary for the efficient optimization of preparative separations. The most accurate method for isotherm determination, frontal analysis, requires a large amount of material and solvent. In contrast, the inverse method simply determines the isotherm of a component from its overloaded peak. The disadvantage of this method is that it requires a prior assumption about the type and equation of the isotherm. The developed unbiased inverse method, however, combines the advantages of frontal analysis and the inverse method. Like the former, it yields isothermal points that are accurate, but like the latter, the isothermal points are determined from an overloaded chromatographic peak, thus requiring much less material and solvent than frontal analysis. This is of great advantage when determining the isotherm of particularly expensive components (e.g., monoclonal antibodies).

## Figures and Tables

**Table 1:** SSR values for each scenario for different numbers of isotherm points.

	Number of Isotherm Points				
Scenario	10	20	40	100	500
1	1.69	2.28 · 10^−2^	$1.60\cdot 10^{-4}$	$1.41\cdot 10^{-7}$	$7.07\cdot 10^{-13}$
2	1.41	$1.40\cdot 10^{-4}$	$1.08\cdot 10^{-6}$	$5.61\cdot 10^{-10}$	$5.84\cdot 10^{-13}$
3	0.46	$1.10\cdot 10^{-4}$	$1.81\cdot 10^{-5}$	$1.98\cdot 10^{-6}$	$2.32\cdot 10^{-8}$
4	4270	156.6	31.05	$2.50\cdot 10^{-4}$	$2.95\cdot 10^{-6}$
5	-	1.54	$2.67\cdot 10^{-3}$	$9.67\cdot 10^{-7}$	$5.73\cdot 10^{-8}$

## Data Availability

The data presented in this study are available on request from the corresponding author.

## Sample Availability

Samples of the compounds are not available from the authors.

## Abbreviations

The following abbreviations are used in this manuscript:

MDPI	Multidisciplinary Digital Publishing Institute
DOAJ	Directory of Open Access Journals
AED	adsorption energy distributions
ECP	Elution by Characteristic Point
FA	Frontal Analysis
FACP	Frontal Analysis by Characteristic Point
HPLC	High Pressure Liquid Chromatography
IM	Inverse Method
RTM	Retention Time Method
SSR	Sum os Square Residuals
